# Recent Insights into Cu-Based Catalytic Sites for the Direct Conversion of Methane to Methanol

**DOI:** 10.3390/molecules27217146

**Published:** 2022-10-22

**Authors:** Min Mao, Lingmei Liu, Zhaohui Liu

**Affiliations:** Institute of Advanced Interdisciplinary Studies, School of Chemistry and Chemical Engineering, Chongqing University, Chongqing 400044, China

**Keywords:** methane to methanol, copper, active site, structure characterization

## Abstract

Direct conversion of methane to methanol is an effective and practical process to improve the efficiency of natural gas utilization. Copper (Cu)-based catalysts have attracted great research attention, due to their unique ability to selectively catalyze the partial oxidation of methane to methanol at relatively low temperatures. In recent decades, many different catalysts have been studied to achieve a high conversion of methane to methanol, including the Cu-based enzymes, Cu-zeolites, Cu-MOFs (metal-organic frameworks) and Cu-oxides. In this mini review, we will detail the obtained evidence on the exact state of the active Cu sites on these various catalysts, which have arisen from the most recently developed techniques and the results of DFT calculations. We aim to establish the structure–performance relationship in terms of the properties of these materials and their catalytic functionalities, and also discuss the unresolved questions in the direct conversion of methane to methanol reactions. Finally, we hope to offer some suggestions and strategies for guiding the practical applications regarding the catalyst design and engineering for a high methanol yield in the methane oxidation reaction.

## 1. Introduction

Nowadays, extensive consumption of energy for transportation, electricity and heat are continuously increasing due to the fast growth of global population and industrial production. However, the primary energy resources are still fossil fuels, even if there are also some alternative renewable energy sources [1,2,3,4,5]. With the sustainability and environmental concerns, considerable efforts have been devoted to the progressive exploration of renewable energy sources. Methane, as a greenhouse gas and an earth-abundant carbon feedstock, is mainly reserved in natural gas [6]. However, the wide application and effective utilization of methane meet the access and transportation challenges. Therefore, the effective catalytic conversion of methane to value-added chemicals has drawn considerable attention [7,8,9,10,11,12].

Because the C−H bonds in methane are quite stable (438.8 kJ/mol), the conversion of methane under moderate reaction conditions is a long-standing challenge [13]. However, its valuable products are readily converted to CO and CO_2_ at high temperatures. The dilemma thus makes the upgrading of methane more complicated [14]. Methanol is thought of as one green raw material for biodiesel and a very important chemical feedstock as well, which can be converted to many different commodities via well-developed techniques [15,16,17]. In the current industrial route, methanol can be formed from methane through a syngas intermediate. However, this indirect process requires high-temperature and high-pressure conditions, requiring large energy consumption [18]. Therefore, the partial oxidation of methane to methanol directly was proposed as the holy grail in chemistry [19]. Many observations and conclusions on the direct conversion of methane to methanol have been reported. Recently, several valuable review articles have already been published, which focused on biological methane oxidation [20] and zeolite-supported metal catalysts for methane conversion [21,22].

Regarding the issues of the methane activation and the overoxidation at high temperatures, considerable efforts have been made in finding and fabricating highly active catalysts that could selectively oxidize methane to methanol at relative moderate conditions [23,24,25,26,27]. Among these investigated catalysts, copper (Cu)-based materials that showed unique catalytic performances have attracted a great deal of interest in the past few decades [21]. Therefore, in this mini review, we elaborate on the recent works concerning the structural studies of the Cu-based reaction centers. In this way, we aim to reveal the structure-determined performances from Cu-based catalytic sites, which include Cu-based enzymes, Cu-zeolites, Cu-MOFs and Cu-oxides. Consequently, based on the knowledge of the structure–performance relationship, we are able to provide some helpful advice for a further catalyst optimization and reaction modification, to finally achieve a high methanol yield from the partial oxidation of methane.

## 2. Cu-Based Enzymes

Methanotrophs, a kind of methane-consuming bacteria living in nature, have the ability to oxidize methane to methanol with their methane monooxygenases (MMOs) at an ambient temperature and pressure. Two kinds of MMOs exist in bacterium; a copper-based particulate membrane-bound enzyme (pMMO) and an iron-based soluble cytoplasmic enzyme (sMMO). The active structure of sMMO is a diiron active site, but the catalytic site of pMMO has remained unclear. Recently, many scientists have devoted their efforts to unveiling the structure of pMMO and its biochemistry on methane oxidation [28,29,30].

### 2.1. The Trinuclearcopper Sites Found in Enzymes

**Figure 1:**
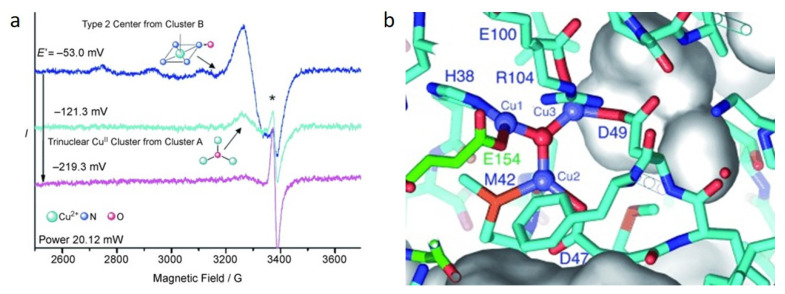
(**a**) EPR spectrum of the copper cluster and (**b**) Trinuclear copper site model in pMMO. Reproduced with permission from ref. [32]. Copyright 2007, Wiley-VCH.

Extensive efforts have been made to reveal the catalytic center of the pMMO enzyme to understand its working mechanism. Because pMMO is an instable complex membrane protein composite, it is rather difficult to be isolated and purified for characterization. In 2004, Chan et al. proposed that the active sites in pMMO were trinuclear copper clusters, which worked for alkane hydroxylation or electron transfer [31]. They then demonstrated the trinuclear copper clusters by an electron paramagnetic resonance spectroscopic (EPR) test, in which the copper ions for alkane hydroxylation were reduced and the intensity of the Cu^II^ EPR signal decreased by increasing the negative potentials (as shown in Figure 1a) [32]. Via a simulation strategy, they confirmed that the EPR signal was assigned to 0.84 Cu^II^ ions at the negative potential of −53.0 mV, which consisted of 0.56 type 2 Cu^II^ ions and 0.29 trinuclear Cu^II^ clusters. At a more negative potential of −121.0 mV, the EPR intensity was only attributed to 0.13 trinuclear Cu^II^ clusters. Therefore, these spectroscopic data showed concrete evidence to confirm the presence of trinuclear copper clusters [32]. In addition, they also modeled a minimized trinuclear copper site computationally, considering the side-chain rotomers, hydrogen bonds, and metal–ligand bonds (Figure 1b) [32]. Via the anaerobic electrospray mass spectrometry, Chen et al. found that the activation of a tri-copper cluster with O_2_/H_2_O_2_ was similar to that of pMMO, also supporting the trinuclear copper cluster theory [33].

### 2.2. The Dicopper Sites Found in Enzymes

The pMMO comprises three polypeptides that are encoded by pmoB, pmoA and pmoC genes. The isolation and purification process should be much elaborate to keep the original statement of the catalytic centers. It is thus a great challenge to determine the nuclearity, ligation and position of the copper sites in pMMO. Advanced characterization techniques were used to deepen our understanding of the catalytic center of the pMMO enzyme. Besides the above-mentioned EPR technique, the extended X-ray absorption fine structure (EXAFS) spectroscopy was also applied in the study and another proposal on the active site was attributed to a dicopper center in PmoB, based on the obtained evidence [34,35].

**Figure 2:**
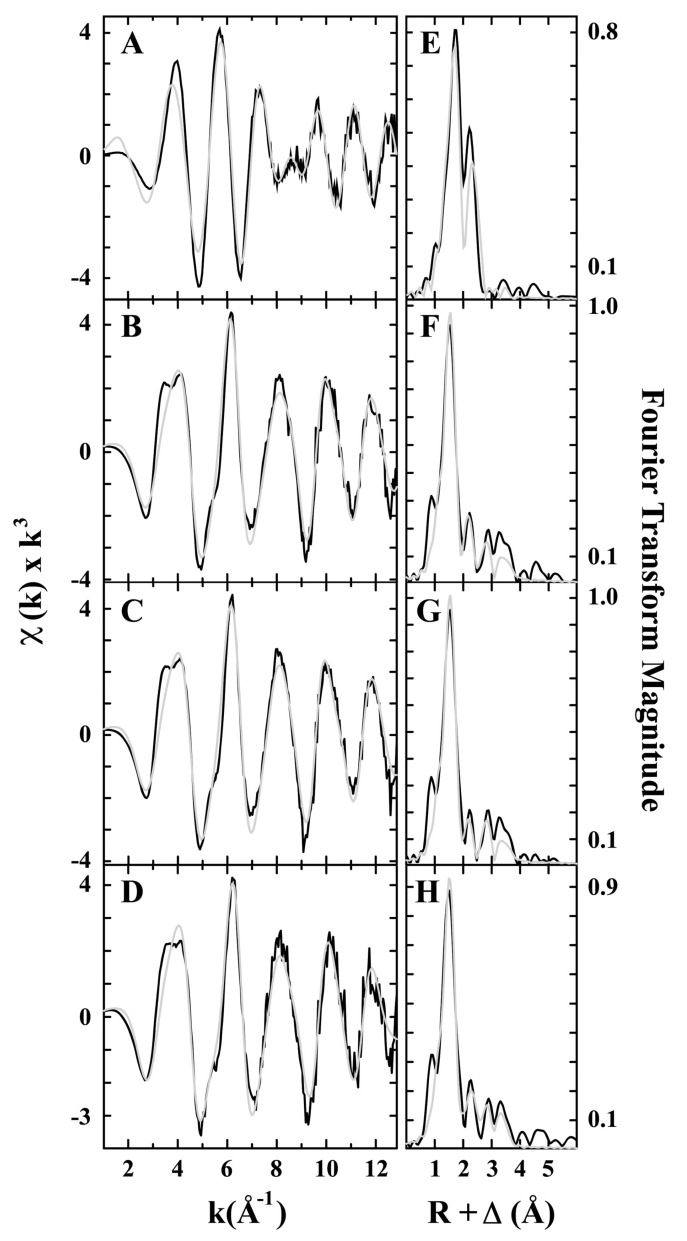
Cu EXAFS fitting analysis for pMMO. Raw data for (**A**) reduced, (**B**) as-isolated, (**C**) Cu-reconstituted, and (**D**) oxidized. The phase-shifted Fourier transform- for (**E**) reduced, (**F**) as-isolated, (**G**) Cu-reconstituted, and (**H**) oxidized. Black spectra are raw EXAFS data and gray spectra are the fitted simulations. Reproduced with permission from ref. [36]. Copyright 2011, American Chemical Society.

In 2003, Rosenzweig and coworkers purified pMMO from methylococcus capsulatus (Bath) and tested its structure by EXAFS [30]. The EXAFS spectra indicated that the purified pMMO contained both Cu^I^ and Cu^II^ oxidation states and a fitted Cu–Cu bond of 2.57 Å, providing direct evidence for a dicopper-containing cluster in pMMO [30]. Later in 2011, the same group gained more detailed results on different treated pMMO samples, as shown in Figure 2 [36]. It was seen that the Fourier transform of the EXAFS data for the samples showed two scattering interactions of the nearest-neighbor ligands at around 2 and 2.5 Å. The EXAFS simulations indicate that each sample contains both Cu−O/N and Cu−Cu ligand environments, and the reduced pMMO has an additional Cu−O/N environment. The bond distance of Cu−O/N ligand is a little longer in the reduced sample (2.02 Å) than the isolated (1.97 Å) and oxidized pMMO (1.96 Å). More importantly, the reduced pMMO has a Cu−Cu bond length of 2.64 Å, which is also longer than that in the as-isolated or oxidized pMMO (2.53 Å) and the Cu-reconstituted pMMO (2.52 Å). Excluding the presence of Cu metal, they proposed that the dinuclear metal sites existed in the tested samples as confirmed by the short Cu−Cu interaction, which was also maintained under the reduction treatment [36]. 

### 2.3. The Monocopper Sites Found in Enzymes

**Figure 3:**
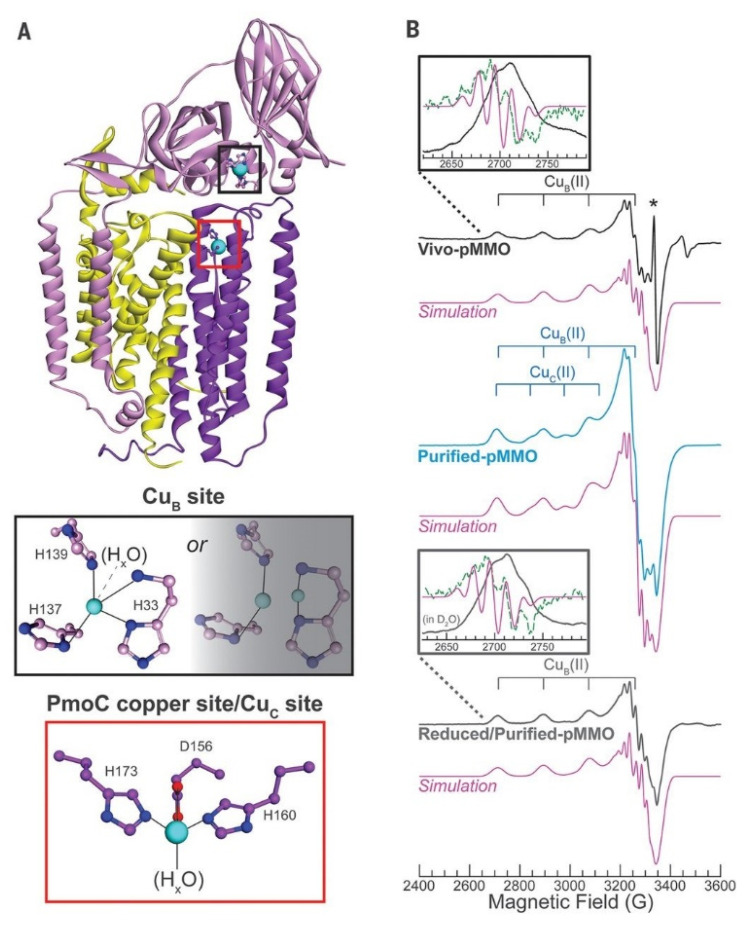
(**A**) Structure of one pMMO with Cu_B_(II) and Cu_C_(II) sites, and (**B**) EPR spectra with simulations of Cu_B_(II) in different pMMOs (in vivo and reduced/purified ones) and Cu_B_(II) plus 0.32 equivalents Cu_C_(II). Reproduced with permission from ref. [37]. Copyright 2019, American Association for the Advancement of Science.

Different from the previous reports, many recent works showed that the monocopper center could also potentially be the catalytic center. In 2019, Ross et al. discovered the evidence of the monocopper sites by advanced characterizations. They probed the pMMO Cu(II) sites with EPR spectroscopy. To circumvent the influence from the loss of copper cofactors in the purification process, they probed the active Cu(II) sites in the whole cells and guaranteed all the Cu(II) sites were present in the in vivo EPR spectrum (Figure 3) [37]. The EPR signal of Cu(II) coordinated by four N equatorial ligands (as illustrated in Figure 3A) was observed in the Vivo-pMMO EPR spectrum of ^15^N and ^63^Cu-enriched M. capsulatus and ^63^Cu hyperfine splitting at 570 MHz. More importantly, two group signals of Cu_B_(II) and Cu_C_(II) in the purified pMMO are consistent with that in Vivo-pMMO EPR spectrum and the simulated one as shown in Figure 3B, which is also consistent in the electron nuclear double resonance (ENDOR) spectroscopy. In addition, they determined the Cu(II)–Cu(II) distances in the purified-pMMO by double electron–electron resonance (DEER) technique. A distance of ~2 nm between Cu_B_(II) and Cu_C_(II) was calculated even if it was too close to be resolved in the direct test. Based on these data, they proposed that two monocopper sites existed in the pMMO enzyme, where one was in the soluble PmoB part and the other was in the membrane-bound PmoC part ~2 nm away. Therefore, a monocopper site was then proposed as the catalytic center in methane oxidation by pMMO [37]. This theory was also corroborated by a native top-down mass spectrometric (nTDMS) technique, which showed the presence of a single copper ion in purified pMMO [38].

By employing EPR and ENDOR (electron-nuclear double resonance) techniques, Hoffman and coworkers investigated the existence of two monocopper sites and the coordination environment [39]. In addition, they applied EPR and ENDOR techniques to examine the copper coordination compounds in pMMO and successfully tracked an internal electron procedure and a Cu(II)−OOH species. They thus confirmed that these advanced tools would be helpful for learning the catalytic mechanism of pMMO [40]. Therefore, these powerful tools provide new approaches to unveil the properties and functionalities of pMMO in the oxidation of methane.

Even though much evidence has already recently been obtained, fundamental insights into the catalytic center in pMMO are still debated. As we discussed in this section, many different copper clusters were discovered and proposed. It remains elusive as to whether purification and crystallization could lead to the copper loss and show the misleading signal of monocopper in characterization. Moreover, Lawton, et al. claimed that the activity of the isolated pMMO from organism was much lower than the original one [41]. Very recently, Change et al. found that one mononuclear copper site, one dicopper site, and some uncovered additional copper clusters presented in one pMMO [42]. Therefore, this long-standing question still needs more cogent evidence to deepen our knowledge.

## 3. Cu-Zeolites

Inspired by enzymology, scientists are taking considerable strides in designing and engineering these similar high-active sites in the catalysis community, which have the ability to convert methane to more valuable products. The crystalline zeolites, which contain various ordered porous frameworks, have been chosen as the potential supports to host the active metal sites [43,44]. In addition, they contain condensing aluminum and silicon tetrahedrons, providing the local coordination environments for copper cations [45]. Therefore, Cu-zeolite is a promising material to mimic the pMMO active site. In this section, we provide an overview of the current understanding on the nature of the catalytic Cu sites supported in zeolite for the direct conversion of methane to methanol.

### 3.1. The Dicopper Sites Supported in Zeolites

In 2005, Groothaert et al. first found that copper supported in zeolites could catalyze methane to methanol, and they observed an obvious decrease in the intensity of a UV–Vis band at 22,700 cm^−1^ of an O_2_-activated catalyst during the reaction process of the methane oxidation. This band is best assigned to the bis(µ-oxo)dicopper core ([Cu_2_-(µ-O)_2_]^2+^), which was first proposed as the catalytic center in the copper hosted in zeolites catalysts. Since then, considerable efforts have been taken to analyze the active sites of Cu-zeolite catalytic systems and improve the methanol yield in the reaction of methane upgrading [21].

**Figure 4:**
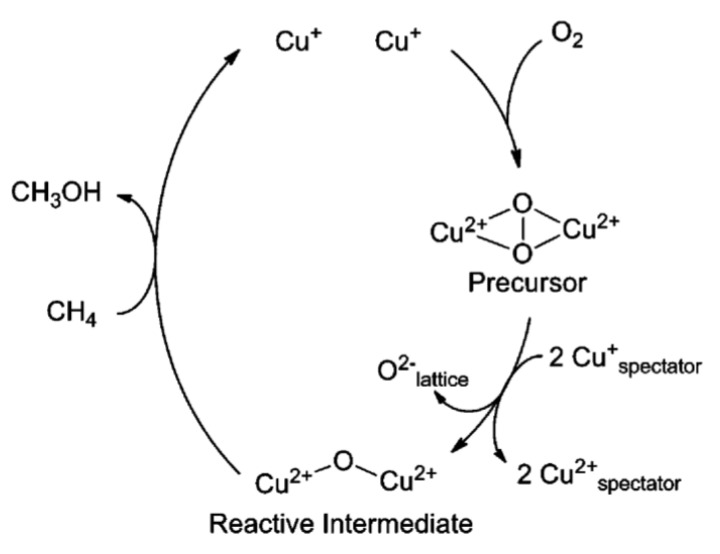
Schematic illustration of the catalytic sites in Cu-ZSM-5 catalyst during the oxidation of methane to methanol. Reproduced with permission from ref. [46]. Copyright 2010, American Chemical Society.

In 2010, Smeets et al. found a UV–Vis absorption band of ~29,000 cm^−1^ when pretreating the Cu-ZSM-5 catalyst with an oxidation procedure. However, this band disappeared gradually with the parallel formation of an absorption band at 22,700 cm^−1^ [46]. Combining the Raman spectra, in which the signals of ν(O-O) and ν(Cu-Cu) were determined at 736 and 269 cm^−1^, respectively, they thus confirmed that the UV–Vis absorption band of 29,000 cm^−1^ was assigned to a peroxo species. The UV–Vis absorption changes demonstrated that the side-on bridged [Cu_2_(O)_2_]^2+^ species transferred to the [Cu_2_O]^2+^ species. Therefore, they proposed that the active center was the [Cu_2_O]^2+^ sites, which was transformed from a precursor of peroxo dicopper(II) species ([Cu_2_(O)_2_]^2+^) in the partial oxidation of methane to methanol (Figure 4) [46].

**Figure 5:**
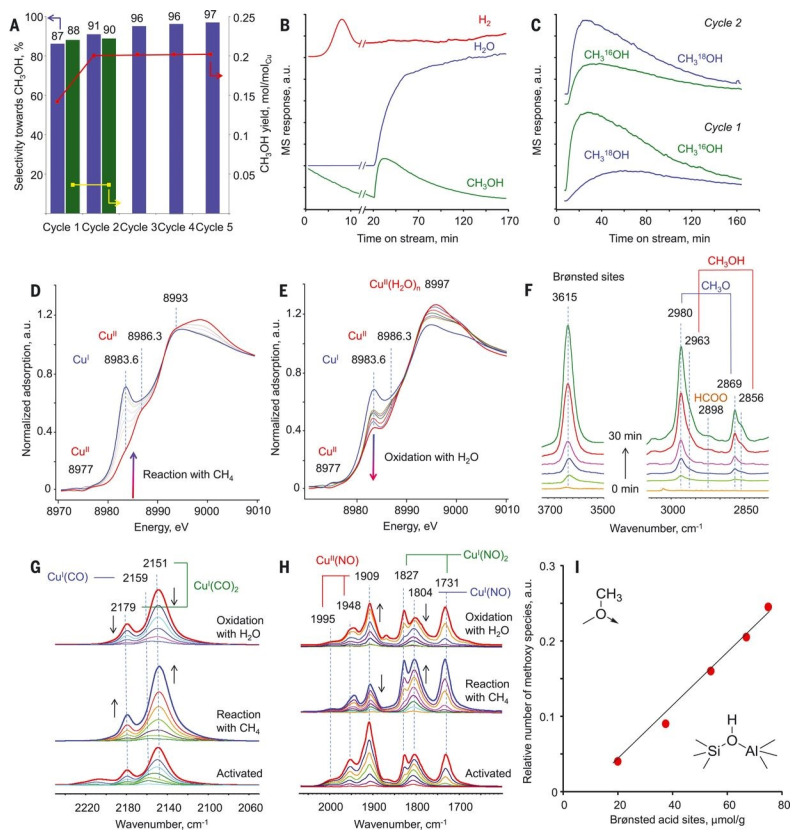
(**A**) Methanol yield and selectivity in methane reaction on Cu-MOR across multiple cycles. (**B**) Mass spectral responses for the reaction cycle. (**C**) Mass spectra of methanol. (**D**) In situ XANES spectra in methane conversion with CuMOR. (**E**) In situ XANES spectra in the reoxidation of Cu-MOR by water. (**F**) In situ FTIR spectra of surface species for the reaction cycle. (**G**) FTIR spectra of CO in different reaction conditions. (**H**) FTIR spectra of NO_2_ in different reaction conditions. (**I**) Relative number of methoxy species vs. number of Brønsted acid sites. Reproduced with permission from ref. [49]. Copyright 2017, American Association for the Advancement of Science.

The dicopper active center is supported by many other reports [46,47]. Vanelderen et al. found that the copper cations supported on Beta zeolite showed similar UV–Vis absorption peaks and Raman features as that of Cu-ZSM-5. Therefore, they believed that the Cu-MOR contained the catalytically active Cu−O−Cu sites located in the 8-MR windows of the side pockets [48]. In 2017, Sushkevich et al. achieved a high methanol yield of 0.204 mole of CH_3_OH per mole of Cu in MOR zeolite [49]. As shown in Figure 5A, the Cu-MOR catalyst shows a very high activity of methane oxidation reaction at a methane pressure of 7 bar at 473 K, and the catalyst can be re-oxidated by water at that temperature. As shown in Figure 5B and C, mass spectra and the isotopic labeling experiments confirmed that water was the only source of oxygen to reactive the spent catalytic centers. Additionally, the x-ray absorption near edge structure (XANES) spectrum of the activated Cu-MOR showed two pre-edge features at 8977 eV and 8986.3 eV of oxidated Cu^II^ and a shoulder at 8983.6 eV of Cu^I^, which were changed with the reaction–reoxidation cycle (Figure 5D,E). Similar behaviors were also obtained in the in situ infrared spectroscopy test (Figure 5F–H). By a further DFT simulation, they finally proposed that the Cu–O–Cu cores were the active centers in Cu-MOR catalyst. On the basis of these results, they demonstrated that the anaerobic oxidation of methane could be achieved on Cu-MOR material with the active dicopper sites [49].

In 2018, Pappas et al. applied multivariate curve resolution analysis of XAS results to exactly quantify the content of active Cu sites in Cu-MOR catalyst and they gained the highest yield of 0.47 mol methanol per mol Cu reported for methane oxidation to methanol over Cu-zeolites. Because the Cu-zeolite catalyst needs to provide two electrons for the oxidation of CH_4_ to CH_3_OH stoichiometrically, they then concluded that the reactive sites were dicopper sites based on the linear relationship between the activated methane amount with the number of Cu sites tested by XAS, i.e., Cu-MOR catalyst activated nearly one methane molecule per two Cu [47].

### 3.2. The Monocopper Sites Supported in Zeolites

**Figure 6:**
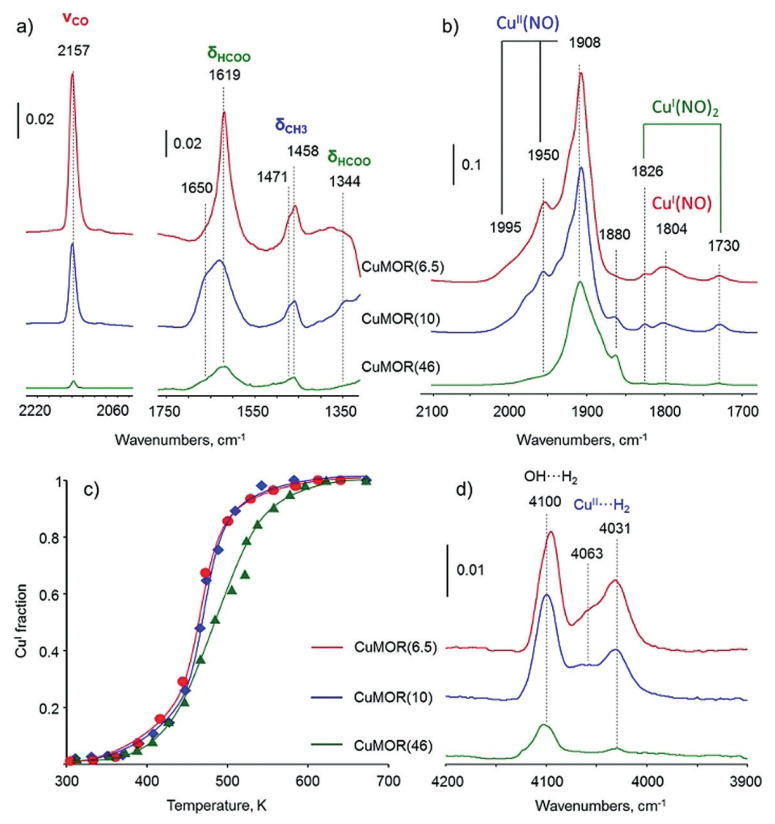
(**a**) In situ FTIR spectra of surface species formed in Cu-MOR samples with different Si/Al ratios (number in the brackets). (**b**) FTIR spectra of NO adsorbed over Cu-MOR. (**c**) Fitted XAS data obtained in the reaction of Cu-MOR with methane. (**d**) FTIR spectra of H_2_ adsorbed on Cu-MOR. Reproduced with permission from ref. [50]. Copyright 2018, Wiley-VCH.

Copper cations are exchanged into the pores of zeolites, and the amount of Cu is thus influenced by the Si/Al ratio of zeolite. Sushkevich et al. found that the catalytic performances varied with the Si/Al ratios in MOR zeolites [50,51]. As shown in Figure 6a, the intensities of IR bands attributed to δ(CH_3_) vibrations decrease in the order of CuMOR(6.5) > CuMOR(10) > CuMOR(46), which is consistent with the Cu amount in MOR. The IR spectra of adsorbed NO were thus applied to assess the copper sites, in which the signal of 1804 cm^−1^ was caused by Cu^I^ mononitrozyl species and the signals at 1826 and 1730 cm^−1^ were assigned to dinitrosyl species (Figure 6b). In situ XANES results revealed that the different copper sites showed different redox properties in the reaction of methane oxidation (Figure 6c). In addition, as shown in Figure 6d, Cu-MOR(6.5) and Cu-MOR(10) showed a stronger interaction with H_2_ than Cu-MOR(46), due to the copper–oxo oligomeric species. On the basis of these data mentioned above, they proposed that the copper monomers ([CuOH]^+^) were presented in the Cu-MOR(46) catalyst [50]. In addition, it was documented that, due to being different from dicopper active sites [49], monomeric copper sites can only be re-oxidated by oxygen [51].

Very recently, Knorpp et al. discovered that the Cu-omega catalyst showed the best reported performance in the stepwise conversion of methane to methanol, in terms of the selectivity and methanol yield. Through the application of anomalous X-ray diffraction (AXPD) and X-ray absorption spectroscopy (XAS), they confirmed that the stabilized paired [CuOH]^+^ monomers in the omega framework contributed to the catalytic property [52]. Ipek et al. also found the presence of bare Cu(II) sites on 6MR of Cu-SSZ-13; however, this proved to be inactive in methane oxidation reaction. However, the adjacent [CuOH]^+^ sites in 8MR of Cu-SSZ-13 would be condensed to generate catalytically active [Cu_2_O_2_]^2+^ and [Cu_2_O]^2+^ sites [53]. Moreover, the relevance between these different active sites was also investigated. Sun et al. found that a key intermediate CuOOH can be formed in both [CuOH]^+^ monomer and [Cu_2_O]^2+^ dimer during the partial oxidation of methane to methanol [54].

### 3.3. The Multiple Copper Clusters Supported in Zeolites

**Figure 7:**
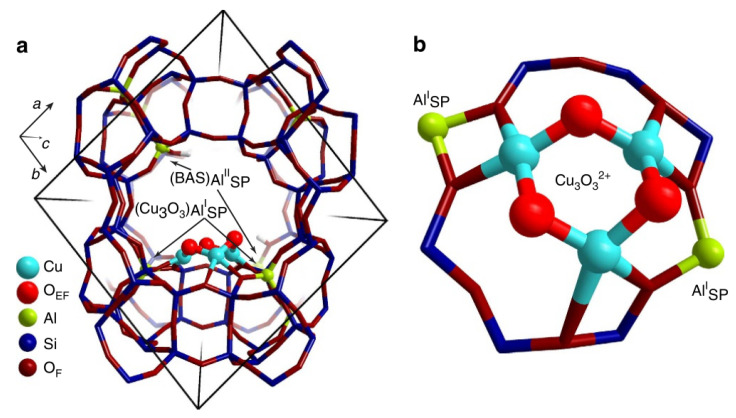
Structure of the trinuclear Cu-oxo cluster in MOR predicted by DFT calculation. In the figure, (**a**) is the main channel of MOR and (**b**) is the MOR side pocket. Reproduced with permission from ref. [55]. Nature Publishing Group.

In addition to the first discovered dicopper core sites, trimeric ([Cu_3_(m-O)_3_]^2+^) sites and even more copper cores are also proposed as the catalytic sites in Cu-zeolite system. In 2015, exploiting the in situ XAS technique, Grundner et al. unveiled that the trinuclear Cu-oxo clusters ([Cu_3_(m-O)_3_]^2+^) were the exclusively single sites in the activated Cu-MOR catalyst under investigation. In combination with ab initio thermodynamic analysis of DFT results, they confirmed that the trinuclear copper clusters were anchored at the windows of the 8-MR MOR side pockets with the connection to two framework Al atoms (Figure 7) [55]. In 2016, Li et al. found that both the binuclear [Cu(µ-O)Cu]^2+^ sites and trinuclear oxygenated [Cu_3_(µ-O)_3_]^2+^ species were presented in ZSM-5 and the trinuclear copper sites were the most stable clusters in Cu/ZSM-5 under calcination conditions [56]. Mahyuddin et al. suggested that the trinuclear copper clusters in MOR and MAZ differed in reactivity in methane conversion based on DFT calculations [57]. Moreover, Palagin et al. proposed the existence of tetra-/pentamer Cu sites of Cu_n_O_n_^2+^ and Cu_n_O_n−1_^2+^ in zeolite pores which exhibited higher relative stability than the smaller clusters [58]. Therefore, we summarized these works together in this paragraph regarding the catalytic center as the multiple copper core clusters. We believe that these multiple copper clusters could be readily formed and may be preferred in zeolites because of the high mobility of Cu atoms under the thermal treatment.

## 4. Cu-MOFs

Metal−organic frameworks (MOFs), as a newly-developed class of porous crystallites, are featured with a high surface area, tailorable structure and diversity. Their periodic networks are built by the self-assembly of metal nodes and organic linkers. The high porosity and organic groups in MOFs enable them to hold guest species in a confined space, especially for metal clusters and metal nanoparticles [59]. Therefore, working as a function in the same way as zeolites, MOFs are another type of porous material to serve as the scaffold backbones for supporting the enzyme-like copper catalytic sites [60].

### 4.1. The Tricopper Sites Anchored in MOFs

**Figure 8:**
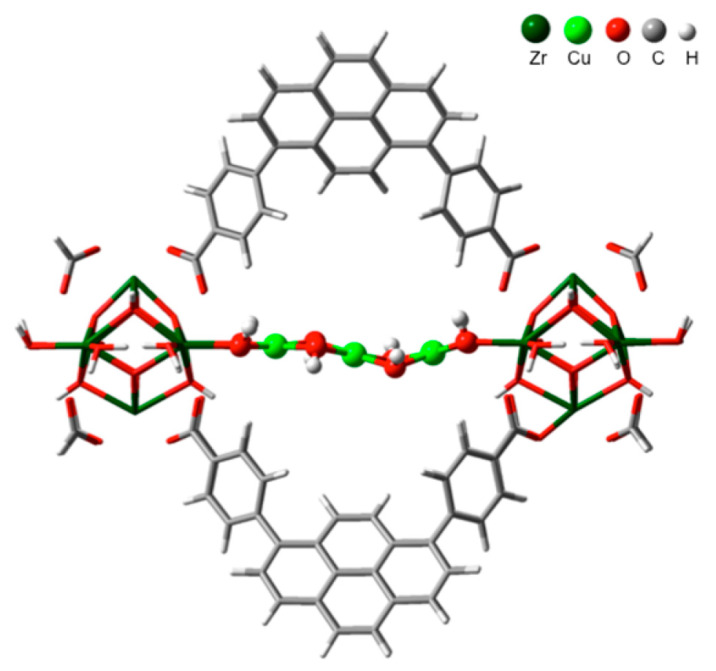
DFT-optimized structure of Cu-NU-1000. Reproduced with permission from ref. [60]. Copyright 2017, American Chemical Society.

In 2017, the Lercher group found that the Cu oxide clusters can be deposited on the ZrO_2_ nodes in a kind of MOF named as NU-1000 using an atomic layer deposition approach [60]. Under an ambient condition, they discovered that the state of the Cu element determined by XAS data contained ∼85% Cu^2+^ and ∼15% Cu^+^, which provided a methanol selectivity of 45−60 C% in the oxidation conversion of methane. Deep analysis of XAS results showed that the Cu atom had a planar four-O-atom coordination structure and an out-of-plane coordinated feature by Jahn−Teller-distorted O atoms. On average, the Cu−Cu distance was calculated as about 2.93 Å and the determined coordination number of Cu was about 1.3. In combination with a DFT study, they proposed that the active center in Cu-NU-1000 was a trimeric Cu-hydroxide-like cluster which was anchored on two nodes of the c-pore of NU-1000 (Figure 8) [60]. Later in 2019, Zheng et al. in the same group prepared mononuclear and dinuclear copper species in NU-1000 through the liquid phase ion exchange method. However, they found that the dinuclear copper sites had the lower energy barrier and better catalytic performance than the mono copper center [61]. These results revealed that the copper sites can also be adjusted in MOF frameworks such as those in zeolite, and the catalytic activity is largely related to the copper clusters.

### 4.2. The Dicopper Sites Anchored in MOFs

**Figure 9:**
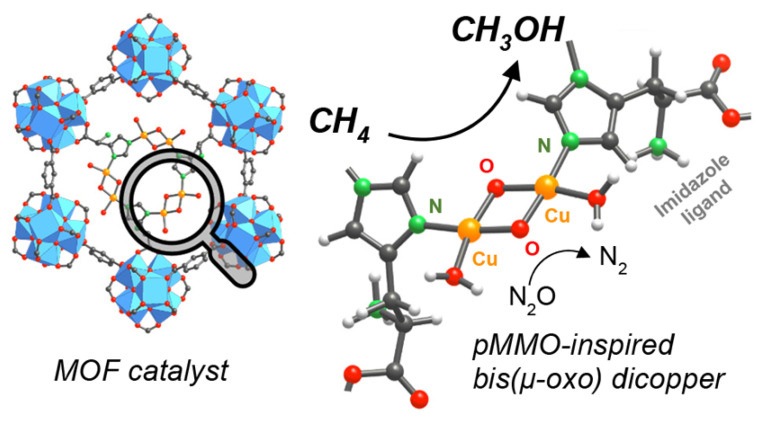
Active sites of Cu/MOF-808 catalyst for partial oxidation of methane to methanol. Reproduced with permission from ref. [62]. Copyright 2018, American Chemical Society.

In 2018, Baek et al. found that the MOF-808 frameworks modified with imidazole units had the capability for subsequent metalation with Cu(I), which thus provided a high selectivity of methanol in methane oxidation at 150 °C. By X-ray absorption near edge structure (XANES) test, they confirmed that Cu atoms were coordinated to N atoms in the imidazole units and the results during the oxidation of Cu(I) to Cu(II) under N_2_O/He exhibited the formation of the active Cu−O species. Furthermore, the Raman peaks observed at ∼560 and ∼640 cm^−1^ were attributed to the vibration of Cu−O bonds in bis(μ-oxo) dicopper species. According to the spectroscopies and DFT calculations, they revealed that the active center is the bis(µ-oxo) dicopper site connected to the N in the imidazole units (Figure 9) [62].

### 4.3. The Mononuclear Copper Sites Anchored in MOFs

**Figure 10:**
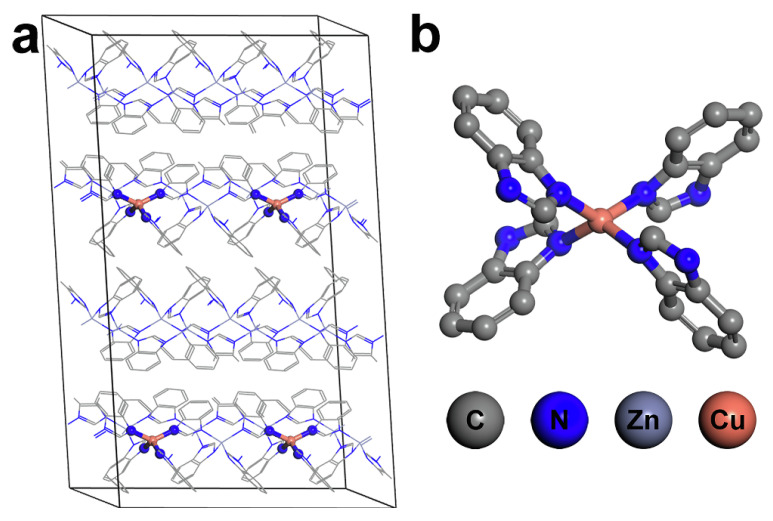
(**a**) Model system and (**b**) an element compound structure of Cu/ZIF-7. Reproduced with permission from ref. [63]. Copyright 2022, Wiley-VCH.

In 2022, Lee et al. embedded Cu^2+^ cation into ZIF-7 and achieved a high partial oxidation activity to the cleavage of the C–H bond in CH_4_. Combining the XAS spectroscopies and DFT calculations, they claimed that the mononuclear CuN_4_ center was the active site in Cu-ZIF-7 for the methane conversion reaction (Figure 10). In addition, they found that the CuN_4_ site could reduce the strong acidity of ZIF-7 as well, which contributed to the high methanol selectivity by avoiding the side-reaction that lead to formic acid forming [63].

## 5. Cu-Oxides

Even though the well-defined porous materials (i.e., zeolites and MOFs) exhibit appropriate features to hold active copper sites for the direct conversion of methane to methanol, the cost for the synthesis of the supporting materials is really high. Searching for other cheap supporting materials is urgently needed in this research area. In 2018, Bozbag et al. found that copper supported on SiO_2_ also showed an effective activity for the partial oxidation of CH_4_ to CH_3_OH in a stepwise way. On UV−Vis spectra, they also observed the absorption band of 22,700 cm^−1^, which was the same as that in Cu-zeolite as mentioned above. Therefore, they assumed that the dicopper [Cu_2_O]^2+^ active sites existed in the Cu/SiO_2_ catalyst which were responsible for the catalytic activity. However, they also observed other unclear absorption bands in the UV−Vis spectra, which may also be the catalytic centers [64].

**Figure 11:**
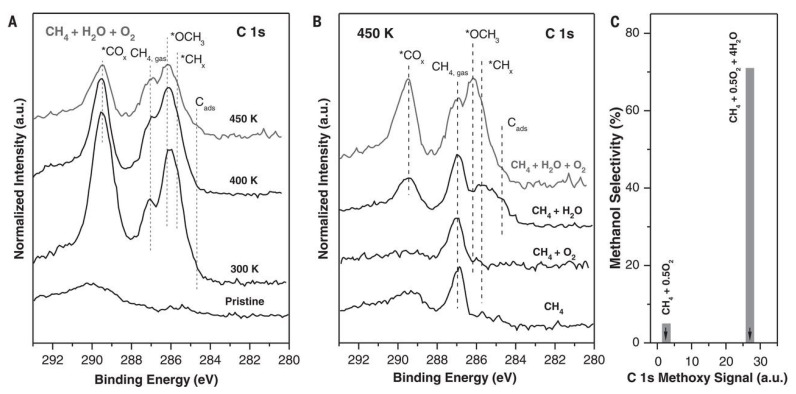
The AP-XPS spectra of C 1s region for (**A**) the CeO_2_/Cu_2_O/Cu(111) surface under a gas mixture of CH_4_, H_2_O and O_2_; and (**B**) exposing CeO_2_/Cu_2_O/Cu(111) catalyst to different gas reactants at 450 K. (**C**) CH_3_OH selectivity generated with and without water. Reproduced with permission from ref. [65]. Copyright 2020, American Association for the Advancement of Science.

In 2020, Liu et al. found that a well-defined CeO_2_/Cu_2_O/Cu(111) structure could selectively catalyze methane to methanol under reaction conditions with methane, water and oxygen [65]. They then revealed the effect of water by ambient-pressure x-ray photoelectron technique (Figure 11). As shown in Figure 11A, the peaks of *CO_x_, CH_4_, *CH_3_O and *CH_x_ are all assigned in the spectra from 300 to 450 K. With the temperatures increased from 300 K to 400 K, the peak for methane is decreased with the presence of a new peak. More importantly, the adsorbed *CH_3_O that is related to methanol is attributed to the peak around 286.2 eV. Furthermore, as shown in Figure 11B, there are very limited amounts of *CH_x_ and *CH_3_O adsorbed on the catalyst surface under pure CH_4_ or CH_4_−O_2_ mixture. In a sharp contrast, the amount of *CH_3_O is largely prompted under a CH_4_−O_2_−H_2_O mixture as shown in Figure 11B. In addition, the peaks of *CH_3_O in AP-XPS are strongly correlated with the formation of methanol in the catalytic tests (Figure 11C). They also demonstrated that water played the similar effect on Cu_2_O/CeO_x_ [66] and Ni/CeO_2_ catalysts [67,68].

## 6. Catalytic Performances of Different Cu-Based Catalysts

As we discussed above, different Cu-based active sites are widely studied for the direct conversion of methane to methanol. The target of these investigations is the same, which is to increase the methanol yield in the oxidation reaction of methane. Numerous investigations have been completed to learn the real active sites in the catalysts, to clarify the structure–performance relationship between the Cu-containing sites and their activity in the reaction of methane to methanol. We summarized the catalytic data in this section, which are listed in detail in Table 1.

The pMMO was first recognized as a kind of Cu-enzyme for the methane conversion, which can work at natural temperatures with air as the oxidant. However, they showed a very low activity and the bacteria need strict conditions to survive [37]. Bioinspired by the enzyme, scientists in chemical engineering are taking considerable strides in designing and engineering these similar high-active sites in the catalysis community. Porous materials (e.g., zeolites and MOFs) are applied to mimic the pMMO structures to support the Cu active sites. In comparison, as shown in Table 1, Cu-zeolites show relative higher activities than Cu-MOFs. In detail, we found that the pretreatment at high temperatures (e.g., 400–500 °C) was needed for Cu-zeolites before the reaction [50]. However, the Cu-MOFs catalysts cannot suffer from these harsh conditions, because of their natural features of the organic ligands. Thereby, based on these results, we assume that the Cu-based active sites are favored at a relatively high temperature. The nonporous oxides also tried to support the Cu sites for the methane conversion reaction. However, they showed even worse catalytic performances than those of Cu-zeolites materials [64].

Comparing these various supports, zeolites seem to be the most promising materials for the catalysts’ engineering. However, the structure of zeolites is also an important factor for determining the catalytic activity of Cu sites. The Cu-MOR was agreed as a good catalyst for the conversion of methane to methanol, in which the Cu active sites were recognized as being located in the 8-MR windows of the side pockets in MOR [50]. In addition, the Cu-CHA catalyst also showed high activity, which may also be caused by its 8-MR pores in CHA zeolite [54]. The exact structures of the Cu active sites in MOR or CHA, as investigated and discussed above, can be both dicopper and monocopper that are responsible for the methane conversion (Table 1).

## 7. Conclusions and Outlook

Methane monooxygenase showed a high activity in the oxidization of methane to methanol at relatively mild conditions. Therefore, the catalytic structure of pMMO attracts much attention in the biochemistry community. The nuclearity of the Cu-based active sites in pMMO have been investigated for decades without any agreement. The advanced EPR, ENDOR and EXAFS characterization techniques have provided many new experimental evidences but the conclusions are still controversial. In addition, it is also debated that the treatment process may damage the structure of pMMO. Therefore, this long-standing question still needs more cogent evidence to deepen our knowledge.

From the inspiration of natural methane monooxygenase, copper cations hosted by zeolite matrix were developed for the direct conversion of methane to methanol reaction. Different to the enzyme, Cu-zeolite has a stable structure that can withstand the harsh reaction conditions. As a consequence, it attracts a great deal of attention from scientists in the research area of chemical engineering. Although a variety of spectroscopies and DFT methods have been used to study Cu-zeolite catalysts, there are still disputes and controversies about the exact state of their catalytic active sites. Another important challenge is that the efficiency of the direct conversion of methane to methanol reaction is extremely low with Cu-zeolite catalysts. Therefore, an in-depth knowledge is urgently required of the structure–performance relationship of Cu-zeolite catalysts on methane oxidation reaction, in order to optimize the catalytic efficiency of Cu-zeolite. Similarly, the studies on Cu-MOFs also did not reach an agreement on the catalytic sites. In addition, another disadvantage for Cu-MOFs is the thermal stability due to the organic linkers in MOFs.

Besides zeolite and MOFs, oxides are also applied to host the Cu active sites. Different to the porous materials, the structures of oxides are relatively simple and easily characterized. The catalytic centers of Cu clusters or nanoparticles are located on the surfaces of supports. The tools for the surface study of the catalyst are thus needed to reveal the structural information. For example, the ambient pressure x-ray photoelectron technique enabled the scientists to obtain quite detailed insights of the Cu-oxides, which showed that the water (possibly the -OH group) promoted the conversion of methane to methanol.

As we discussed above, various supports have been studied to host the Cu-based catalytic sites for the direct conversion of methane to methanol. The target of these investigations is the same, which is to increase the methanol yield in the oxidation reaction of methane. However, from the reported results, the efficiency of the studied reaction is still extremely low. We think it might still be a long way to attain a satisfactory methanol yield in methane conversion. As a fundamental element of the study, it is an essential path to reveal the catalytic centers of catalysts, not limited to Cu-based materials, via advanced characterization techniques. Then, this knowledge will guide the practical applications regarding the catalyst design and engineering.

## Figures and Tables

**Table 1:** Catalytic performances of different catalysts for the direct conversion of methane to methanol.

Catalyst	Active Sites	Oxidant	Activation Temperature (°C)	Reaction Temperature (°C)	Reaction Rate ^a^ (mol_CH3OH_/mol_Cu_)	Ref.
pMMO	Dicopper	Air	37	37	5.3 nmol/(min mg)	[36]
pMMO	Monocopper	Air	45	45	9 µM/h	[37]
pMMO	Monocopper	Air	35	35	17 µM/12 h	[37]
Cu-ZSM-5	[Cu_2_-(µ-O)_2_]^2+^[Cu_2_-(µ-O)_2_]^2+^	O_2_	450	100	0.029/cycle	[21]
Cu-MOR		O_2_	450	100	0.018/cycle	[21]
Cu/SiO_2_	/	O_2_	450	100	0.003/cycle	[21]
Cu-ZSM-5	[Cu_2_O]^2+^	O_2_	450	100	/	[46]
Cu-MOR	[Cu_2_O]^2+^	H_2_O	400	200	0.204/cycle	[49]
Cu-MOR	[Cu_2_O]^2+^	O_2_	500	200	0.47/cycle	[47]
Cu-MOR(6.5)	[Cu_2_O]^2+^	O_2_	400	200	0.142cycle	[50]
Cu-MOR(10)	[Cu_2_O]^2+^	O_2_	400	200	0.216/cycle	[50]
Cu-MOR(46)	[CuOH]^+^	O_2_	400	200	0.316/cycle	[50]
Cu-MOR(6.5)	[Cu_2_O]^2+^	H_2_O	400	200	0.204/cycle	[50]
Cu-MOR(10)	[Cu_2_O]^2+^	H_2_O	400	200	0.187/cycle	[50]
Cu-MOR(46)	[CuOH]^+^	H_2_O	400	200	0/cycle	[50]
Cu-omega	[CuOH]^+^	O_2_	450	200	0.22/cycle	[52]
Cu-CHA	CuOOH	O_2_/H_2_O	300	300	0.543/h	[54]
Cu-NU-1000	Tricopper	O_2_	200	150	17.7 µmol/g	[60]
Cu/MOF-808	Dicopper	N_2_O	150	150	71.8 µmol/g	[62]
Cu-ZIF-7	Monocopper	H_2_O_2_	50	50	~100 µmol/g	[63]
Cu/SiO_2_	[Cu_2_O]^2+^	O_2_	500	200	0.016/cycle	[64]
Cu/SiO_2_	[Cu_2_O]^2+^	O_2_	800	200	0.037/cycle	[64]
CeO_2_/Cu_2_O/Cu	/	O_2_/H_2_O	177	177	70% Sel.^b^	[65]

^a^, for the stepwise reaction, the reaction rate is presented with per cycle. ^b^, the selectivity of methanol is 70%.

## Data Availability

Not applicable.

## References

[1] Primo A., Garcia H. (2014). Zeolites as catalysts in oil refining. Chem. Soc. Rev..

[2] Taarning E., Osmundsen C.M., Yang X., Voss B., Andersen S.I., Christensen C.H. (2011). Zeolite-catalyzed biomass conversion to fuels and chemicals. Energy Environ. Sci..

[3] Shaner M.R., Davis S.J., Lewis N.S., Caldeira K. (2018). Geophysical constraints on the reliability of solar and wind power in the United States. Energ. Environ. Sci..

[4] Li C., Cao Q., Wang F., Xiao Y., Li Y., Delaunay J.J., Zhu H. (2018). Engineering graphene and TMDs based van der Waals heterostructures for photovoltaic and photoelectrochemical solar energy conversion. Chem. Soc. Rev..

[5] Yangcheng R.X., Ran J.S., Liu Z.H., Cui Y.T., Wang J.J. (2022). Phosphoric acid-modified commercial kieselguhr supported palladium nanoparticles as efficient catalysts for low-temperature hydrodeoxygenation of lignin derivatives in water. Green Chem..

[6] Aasberg-Petersen K., Dybkjaer I., Ovesen C.V., Schjodt N.C., Sehested J., Thomsen S.G. (2011). Natural gas to synthesis gas—Catalysts and catalytic processes. J. Nat. Gas Sci. Eng..

[7] Guo X., Fang G., Li G., Ma H., Fan H., Yu L., Ma C., Wu X., Deng D., Wei M. (2014). Direct, nonoxidative conversion of methane to ethylene, aromatics, and hydrogen. Science.

[8] Qi G., Davies T.E., Nasrallah A., Sainna M.A., Howe A.G.R., Lewis R.J., Quesne M., Catlow C.R.A., Willock D.J., He Q. (2022). Au-ZSM-5 catalyses the selective oxidation of CH4 to CH3OH and CH3COOH using O_2_. Nat. Catal..

[9] Cui X., Huang R., Deng D. (2021). Catalytic conversion of C1 molecules under mild conditions. EnergyChem.

[10] Zichittella G., Perez-Ramirez J. (2021). Status and prospects of the decentralised valorisation of natural gas into energy and energy carriers. Chem. Soc. Rev..

[11] Razdan N.K., Bhan A. (2020). Carbidic Mo is the sole kinetically-relevant active site for catalytic methane dehydroaromatization on Mo/H-ZSM-5. J. Catal..

[12] Morejudo S.H., Zanon R., Escolastico S., Yuste-Tirados I., Malerod-Fjeld H., Vestre P.K., Coors W.G., Martinez A., Norby T., Serra J.M. (2016). Direct conversion of methane to aromatics in a catalytic co-ionic membrane reactor. Science.

[13] Periana R.A., Taube D.J., Evitt E.R., Loffler D.G., Wentrcek P.R., Voss G., Masuda T. (1993). A mercury-catalyzed, high-yield system for the oxidation of methane to methanol. Science.

[14] Luo L., Luo J., Li H., Ren F., Zhang Y., Liu A., Li W.X., Zeng J. (2021). Water enables mild oxidation of methane to methanol on gold single-atom catalysts. Nat. Commun..

[15] Olsbye U., Svelle S., Bjorgen M., Beato P., Janssens T.V., Joensen F., Bordiga S., Lillerud K.P. (2012). Conversion of methanol to hydrocarbons: How zeolite cavity and pore size controls product selectivity. Angew. Chem. Int. Ed..

[16] Hua J., Dong X., Wang J., Chen C., Shi Z., Liu Z., Han Y. (2020). Methanol-to-olefin conversion over small-pore DDR zeolites: Tuning the propylene selectivity via the olefin-based catalytic cycle. ACS Catal..

[17] Liu Z., Dong X., Zhu Y., Emwas A.-H., Zhang D., Tian Q., Han Y. (2015). Investigating the influence of mesoporosity in zeolite Beta on its catalytic performance for the conversion of methanol to hydrocarbons. ACS Catal..

[18] Usman M., Daud W.M.A.W. (2015). Recent advances in the methanol synthesis via methane reforming processes. RSC Adv..

[19] Kosinov N., Hensen E.J.M. (2020). Reactivity, Selectivity, and Stability of Zeolite-Based Catalysts for Methane Dehydroaromatization. Adv. Mater..

[20] Koo C.W., Rosenzweig A.C. (2021). Biochemistry of aerobic biological methane oxidation. Chem. Soc. Rev..

[21] Newton M.A., Knorpp A.J., Sushkevich V.L., Palagin D., van Bokhoven J.A. (2020). Active sites and mechanisms in the direct conversion of methane to methanol using Cu in zeolitic hosts: A critical examination. Chem. Soc. Rev..

[22] Kiani D., Sourav S., Tang Y., Baltrusaitis J., Wachs I.E. (2021). Methane activation by ZSM-5-supported transition metal centers. Chem. Soc. Rev..

[23] Fan Y.Y., Zhou W.C., Qiu X.Y., Li H.D., Jiang Y.H., Sun Z.H., Han D.X., Niu L., Tang Z.Y. (2021). Selective photocatalytic oxidation of methane by quantum-sized bismuth vanadate. Nat. Sustain..

[24] Feng N., Lin H., Song H., Yang L., Tang D., Deng F., Ye J. (2021). Efficient and selective photocatalytic CH4 conversion to CH_3_OH with O_2_ by controlling overoxidation on TiO_2_. Nat. Commun..

[25] Agarwal N., Freakley S.J., McVicker R.U., Althahban S.M., Dimitratos N., He Q., Morgan D.J., Jenkins R.L., Willock D.J., Taylor S.H. (2017). Aqueous Au-Pd colloids catalyze selective CH_4_ oxidation to CH_3_OH with O_2_ under mild conditions. Science.

[26] Jin Z., Wang L., Zuidema E., Mondal K., Zhang M., Zhang J., Wang C., Meng X., Yang H., Mesters C. (2020). Hydrophobic zeolite modification for in situ peroxide formation in methane oxidation to methanol. Science.

[27] Fjermestad T., Genest A., Li W., Mestl G., Roesch N. (2017). Surface Reactivity of the Vanadium Phosphate Catalyst for the Oxidation of Methane. Top. Catal..

[28] Peng W., Qu X., Shaik S., Wang B. (2021). Deciphering the oxygen activation mechanism at the CuC site of particulate methane monooxygenase. Nat. Catal..

[29] Yu S.S.F., Chen K.H.C., Tseng M.Y.H., Wang Y.S., Tseng C.F., Chen Y.J., Huang D.S., Chan S.I. (2003). Production of high-quality particulate methane monooxygenase in high yields from Methylococcus capsulatus (Bath) with a hollow-fiber membrane bioreactor. J. Bacteriol..

[30] Lieberman R.L., Shrestha D.B., Doan P.E., Hoffman B.M., Stemmler T.L., Rosenzweig A.C. (2003). Purified particulate methane monooxygenase from Methylococcus capsulatus (Bath) is a dimer with both mononuclear copper and a copper-containing cluster. Proc. Natl. Acad. Sci. USA.

[31] Chan S.I., Chen K.H.C., Yu S.S.F., Chen C.L., Kuo S.S.J. (2004). Toward delineating the structure and function of the particulate methane monooxygenase from methanotrophic bacteria. Biochemistry.

[32] Chan S.I., Wang V.C., Lai J.C., Yu S.S., Chen P.P., Chen K.H., Chen C.L., Chan M.K. (2007). Redox potentiometry studies of particulate methane monooxygenase: Support for a trinuclear copper cluster active site. Angew. Chem. Int. Ed..

[33] Chen Y.H., Wu C.Q., Sung P.H., Chan S.I., Chen P.P.Y. (2020). Turnover of a Methane Oxidation Tricopper Cluster Catalyst: Implications for the Mechanism of the Particulate Methane Monooxygenase (pMMO). Chemcatchem.

[34] Lieberman R.L., Rosenzweig A.C. (2005). Crystal structure of a membrane-bound metalloenzyme that catalyses the biological oxidation of methane. Nature.

[35] Balasubramanian R., Smith S.M., Rawat S., Yatsunyk L.A., Stemmler T.L., Rosenzweig A.C. (2010). Oxidation of methane by a biological dicopper centre. Nature.

[36] Smith S.M., Rawat S., Telser J., Hoffman B.M., Stemmler T.L., Rosenzweig A.C. (2011). Crystal Structure and Characterization of Particulate Methane Monooxygenase from Methylocystis species Strain M. Biochemistry.

[37] Ross M.O., MacMillan F., Wang J., Nisthal A., Lawton T.J., Olafson B.D., Mayo S.L., Rosenzweig A.C., Hoffman B.M. (2019). Particulate methane monooxygenase contains only mononuclear copper centers. Science.

[38] Ro S.Y., Schachner L.F., Koo C.W., Purohit R., Remis J.P., Kenney G.E., Liauw B.W., Thomas P.M., Patrie S.M., Kelleher N.L. (2019). Native top-down mass spectrometry provides insights into the copper centers of membrane-bound methane monooxygenase. Nat. Commun..

[39] Jodts R.J., Ross M.O., Koo C.W., Doan P.E., Rosenzweig A.C., Hoffman B.M. (2021). Coordination of the Copper Centers in Particulate Methane Monooxygenase: Comparison between Methanotrophs and Characterization of the CuC Site by EPR and ENDOR Spectroscopies. J. Am. Chem. Soc..

[40] Davydov R., Herzog A.E., Jodts R.J., Karlin K.D., Hoffman B.M. (2022). End-On Copper(I) Superoxo and Cu(II) Peroxo and Hydroperoxo Complexes Generated by Cryoreduction/Annealing and Characterized by EPR/ENDOR Spectroscopy. J. Am. Chem. Soc..

[41] Lawton T.J., Rosenzweig A.C. (2016). Methane-Oxidizing Enzymes: An Upstream Problem in Biological Gas-to-Liquids Conversion. J. Am. Chem. Soc..

[42] Chang W.H., Lin H.H., Tsai I.K., Huang S.H., Chung S.C., Tu I.P., Yu S.S., Chan S.I. (2021). Copper Centers in the Cryo-EM Structure of Particulate Methane Monooxygenase Reveal the Catalytic Machinery of Methane Oxidation. J. Am. Chem. Soc..

[43] Liu Z., Hua Y., Wang J., Dong X., Tian Q., Han Y. (2017). Recent progress in the direct synthesis of hierarchical zeolites: Synthetic strategies and characterization methods. Mater. Chem. Front..

[44] Del Campo P., Martinez C., Corma A. (2021). Activation and conversion of alkanes in the confined space of zeolite-type materials. Chem. Soc. Rev..

[45] Chai Y., Dai W., Wu G., Guan N., Li L. (2021). Confinement in a Zeolite and Zeolite Catalysis. Acc. Chem. Res..

[46] Smeets P.J., Hadt R.G., Woertink J.S., Vanelderen P., Schoonheydt R.A., Sels B.F., Solomon E.I. (2010). Oxygen precursor to the reactive intermediate in methanol synthesis by Cu-ZSM-5. J. Am. Chem. Soc..

[47] Pappas D.K., Martini A., Dyballa M., Kvande K., Teketel S., Lomachenko K.A., Baran R., Glatzel P., Arstad B., Berlier G. (2018). The nuclearity of the active site for methane to methanol conversion in Cu-mordenite: A quantitative assessment. J. Am. Chem. Soc..

[48] Vanelderen P., Snyder B.E., Tsai M.L., Hadt R.G., Vancauwenbergh J., Coussens O., Schoonheydt R.A., Sels B.F., Solomon E.I. (2015). Spectroscopic definition of the copper active sites in mordenite: Selective methane oxidation. J. Am. Chem. Soc..

[49] Sushkevich V.L., Palagin D., Ranocchiari M., van Bokhoven J.A. (2017). Selective anaerobic oxidation of methane enables direct synthesis of methanol. Science.

[50] Sushkevich V.L., Palagin D., van Bokhoven J.A. (2018). The effect of the active-site structure on the activity of copper mordenite in the aerobic and anaerobic conversion of methane into methanol. Angew. Chem. Int. Ed..

[51] Sushkevich V.L., Verel R., van Bokhoven J.A. (2020). Pathways of methane transformation over copper-exchanged mordenite as revealed by in situ NMR and IR spectroscopy. Angew. Chem. Int. Ed..

[52] Knorpp A.J., Pinar A.B., Baerlocher C., McCusker L.B., Casati N., Newton M.A., Checchia S., Meyet J., Palagin D., Bokhoven J.A. (2021). Paired copper monomers in zeolite omega: The active site for methane-to-methanol conversion. Angew. Chem. Int. Ed..

[53] Ipek B., Wulfers M.J., Kim H., Goltl F., Hermans I., Smith J.P., Booksh K.S., Brown C.M., Lobo R.F. (2017). Formation of [Cu_2_O_2_]^2+^ and [Cu_2_O]^2+^ toward C-H bond activation in Cu-SSZ-13 and Cu-SSZ-39. ACS Catal..

[54] Sun L.L., Wang Y., Wang C.M., Xie Z.K., Guan N.J., Li L.D. (2021). Water-involved methane-selective catalytic oxidation by dioxygen over copper zeolites. Chem.

[55] Grundner S., Markovits M.A., Li G., Tromp M., Pidko E.A., Hensen E.J., Jentys A., Sanchez-Sanchez M., Lercher J.A. (2015). Single-site trinuclear copper oxygen clusters in mordenite for selective conversion of methane to methanol. Nat. Commun..

[56] Li G., Vassilev P., Sanchez-Sanchez M., Lercher J.A., Hensen E.J.M., Pidko E.A. (2016). Stability and reactivity of copper oxo-clusters in ZSM-5 zeolite for selective methane oxidation to methanol. J. Catal..

[57] Mahyuddin M.H., Tanaka T., Shiota Y., Staykov A., Yoshizawa K. (2018). Methane Partial Oxidation over [Cu2(μ-O)]2+ and [Cu3(μ-O)3]2+ Active Species in Large-Pore Zeolites. ACS Catal..

[58] Palagin D., Knorpp A.J., Pinar A.B., Ranocchiari M., van Bokhoven J.A. (2017). Assessing the relative stability of copper oxide clusters as active sites of a CuMOR zeolite for methane to methanol conversion: Size matters?. Nanoscale.

[59] Yang Q., Xu Q., Jiang H.L. (2017). Metal-organic frameworks meet metal nanoparticles: Synergistic effect for enhanced catalysis. Chem. Soc. Rev..

[60] Ikuno T., Zheng J., Vjunov A., Sanchez-Sanchez M., Ortuno M.A., Pahls D.R., Fulton J.L., Camaioni D.M., Li Z., Ray D. (2017). Methane oxidation to methanol catalyzed by Cu-oxo clusters stabilized in NU-1000 metal-organic framework. J. Am. Chem. Soc..

[61] Zheng J., Ye J., Ortuno M.A., Fulton J.L., Gutierrez O.Y., Camaioni D.M., Motkuri R.K., Li Z., Webber T.E., Mehdi B.L. (2019). Selective Methane Oxidation to Methanol on Cu-Oxo Dimers Stabilized by Zirconia Nodes of an NU-1000 Metal-Organic Framework. J. Am. Chem. Soc..

[62] Baek J., Rungtaweevoranit B., Pei X., Park M., Fakra S.C., Liu Y.S., Matheu R., Alshmimri S.A., Alshehri S., Trickett C.A. (2018). Bioinspired Metal-Organic Framework Catalysts for Selective Methane Oxidation to Methanol. J. Am. Chem. Soc..

[63] Lee H., Kwon C., Keum C., Kim H.E., Lee H., Han B., Lee S.Y. (2022). Methane partial oxidation by monomeric Cu active center confined on ZIF-7. Chem. Eng. J..

[64] Bozbag S.E., Sot P., Nachtegaal M., Ranocchiari M., van Bokhoven J.A., Mesters C. (2018). Direct stepwise oxidation of methane to methanol over Cu–SiO_2_. ACS Catal..

[65] Liu Z., Huang E., Orozco I., Liao W., Palomino R.M., Rui N., Duchon T., Nemsak S., Grinter D.C., Mahapatra M. (2020). Water-promoted interfacial pathways in methane oxidation to methanol on a CeO_2_-Cu_2_O catalyst. Science.

[66] Zuo Z., Ramirez P.J., Senanayake S.D., Liu P., Rodriguez J.A. (2016). Low-Temperature Conversion of Methane to Methanol on CeOx/Cu2O Catalysts: Water Controlled Activation of the C-H Bond. J. Am. Chem. Soc..

[67] Lustemberg P.G., Palomino R.M., Gutierrez R.A., Grinter D.C., Vorokhta M., Liu Z., Ramirez P.J., Matolin V., Ganduglia-Pirovano M.V., Senanayake S.D. (2018). Direct conversion of methane to methanol on Ni-ceria surfaces: Metal-support interactions and water-enabled catalytic conversion by site blocking. J. Am. Chem. Soc..

[68] Huang E., Orozco I., Ramirez P.J., Liu Z., Zhang F., Mahapatra M., Nemsak S., Senanayake S.D., Rodriguez J.A., Liu P. (2021). Selective Methane Oxidation to Methanol on ZnO/Cu_2_O/Cu(111) Catalysts: Multiple Site-Dependent Behaviors. J. Am. Chem. Soc..

